# “It’s not all about the disease”: do treatment and socioeconomic status affect perceived impact and satisfaction of patients treated for cutaneous leishmaniasis?

**DOI:** 10.1590/0037-8682-0253-2022

**Published:** 2023-02-20

**Authors:** Carolina Di Pietro Carvalho, João Gabriel Guimarães Luz, Amanda Gabriela de Carvalho, Renata Di Pietro Carvalho, Herton Helder Rocha Pires, João Victor Leite Dias

**Affiliations:** 1 Universidade Federal dos Vales do Jequitinhonha e Mucuri, Programa de Pós-graduação em Saúde, Sociedade e Ambiente, Diamantina, MG, Brasil.; 2 Secretaria Estadual de Saúde, Superintendência Regional de Saúde, Diamantina, MG, Brasil.; 3 Universidade Federal de Rondonópolis, Faculdade de Ciências da Saúde, Rondonópolis, MT, Brasil.; 4 Universidade Federal dos Vales do Jequitinhonha e Mucuri, Faculdade de Medicina do Mucuri, Teófilo Otoni, MG, Brasil.

**Keywords:** Cutaneous leishmaniasis, Neglected diseases, Patient satisfaction, Quality of life

## Abstract

**Background:**

This cross-sectional study compared the general impact of cutaneous leishmaniasis (CL) and patient satisfaction with treatment and health services as perceived by those undergoing different therapeutic regimens in an endemic region in South-Eastern Brazil. We also investigated the factors associated with both outcomes (general impact and satisfaction).

**Methods:**

We included 84 patients with CL treated between 2018 and 2019 with intravenous meglumine antimoniate, liposomal amphotericin B, or intralesional meglumine antimoniate therapy. Data were collected through interviews that assessed sociodemographic characteristics, comorbidity status, access and use of health services for CL diagnosis and treatment, and the items of the Cutaneous Leishmaniasis Impact Questionnaire (CLIQ). The CLIQ is a psychometric questionnaire previously validated to assess the general impact of CL on patient satisfaction with treatment and health services. Multivariate logistic regression analysis was used to identify the factors associated with high CL impact and low patient satisfaction.

**Results:**

The general impact of CL and patient satisfaction with treatment and health services were not significantly associated with the therapeutic regimen. High CL impact was associated with low family income (odds ratio [OR]:3.3; 95% confidence interval [CI]:1.0-10.3), occurrence of complications/adverse effects during treatment (OR:7.7; 95%CI:2.4-25.6), and additional costs during diagnosis and/or treatment (OR:12.1; 95% CI:2.8-52.4). Low satisfaction was associated with high disease impact (OR: 9.5; 95% CI:2.7-33.9), occurrence of complications/adverse effects (OR:4.2; 95% CI:1.3-13.0), and high family income (OR:7.1; 95%CI:1.7-28.2).

**Conclusions:**

Our data support public health policies aimed at reducing the impact of CL and its treatment as well as the use of therapy with fewer adverse effects.

## INTRODUCTION

Cutaneous leishmaniasis (CL) is a vector-borne disease caused by protozoa of the genus *Leishmania*. Clinically, CL is characterized by the involvement of cutaneous (cutaneous leishmaniasis) and/or mucosal tissues (mucocutaneous leishmaniasis), with a high risk of physical deformity^1^. Although death due to CL is rare, irreversible deformation has a negative social and economic impact on a patient’s quality of life^2^ ^,^ ^3^. This impact is a consequence of social stigma and post-infection psychological issues and is characterized by the loss of school opportunities and work capacity, expressed as disability-adjusted life years (DALY)^2^ ^,^ ^4^. Brazil is among the countries with the highest DALY sowing to CL^5^. Annually, over 26,000 new CL cases are reported nationwide^1^, mainly among individuals with low socioeconomic status^6^. The negative impact of CL on individuals with low socioeconomic status is usually greater due to the difficulty of access and accessibility to health services for diagnosis and treatment^7^ ^,^ ^8^ ^,^ ^9^.

The first-line treatment for CL currently recommended by the Brazilian Ministry of Health is systemic therapy with intravenous administration of meglumine antimoniate (IV-MA), a pentavalent antimonial^1^. Although therapy with IV-MA results in high cure rates, it has significant adverse events and requires at least 20 days of outpatient administration^1^ ^,^ ^10^ ^,^ ^11^.Intravenous administration of amphotericin B in deoxycholate or, more frequently, liposomal IV-LAB formulations are the second-line therapies. IV-LAB is recommended to treat patients who experience relapse; individuals with kidney, heart, and liver failure, pregnant women, and people aged over 50 years^1^. It has potent leishmanicidal action and fewer adverse events than that of antimonials. However, the need for parenteral administration in hospitals and its high cost may limit its use^12^.

Given the afore mentioned limitations and following the recommendations of the World Health Organization^10^, the Brazilian Ministry of Health has incorporated the intralesional administration of meglumine antimoniate (IL-MA) as an alternative treatment for CL in 2017^1^. In addition, IL-MA is performed in a maximum of three applications spaced in time, which results in less systemic absorption and fewer adverse effects^1^. These characteristics make the treatment simpler, safer, and more effective^13^. Consequently, a decrease in treatment-associated operational difficulties is expected in both patients and health services^14^. This is particularly desirable because the first-line treatment for CL in Brazil is mainly performed in basic care units (BCUs). BCUs are primary healthcare centers with limited resources for managing comorbidities and, thus, face difficulties monitoring and managing the adverse effects of CL treatment^15^.

The impact of CL on patients' lives and satisfaction with treatment and health services has recently been studied in Brazil^16^. Such investigations are useful for planning public health policies aimed at improving clinical management and assistance offered by health services^17^. However, no studies have specifically addressed the impact and satisfaction with CL treatment as perceived by patients undergoing different therapeutic regimens in Brazil, including the recently implemented IL-MA therapy. Thus, we attempted to address this topic in patients affected by CL in an area endemic to the disease in the Brazilian state of Minas Gerais. In addition, we investigated factors associated with the high impact of CL and low satisfaction with health services during treatment.

## METHODS

### Design and study area

This was an epidemiological, descriptive, cross-sectional study carried out by administering two questionnaires among individuals treated for CL between 2018 and 2019 using different therapeutic schemes in municipalities under the jurisdiction of the Regional Health Superintendence of Diamantina (RHS/Diamantina).

RHS/Diamantina is one of the 28 regional health superintendencies that constitutes the administrative and health organization of the state of Minas Gerais in South-Eastern Brazil. RHS/Diamantina comprises 33 municipalities, with a total surface area of 33,733,286 km². Among the municipalities, 29 were located in the Jequitinhonha mesoregion and four in the central mesoregion of the state. In particular, municipalities within the extended mesoregion of Jequitinhonha have historically reported one of the worst demographic and socioeconomic indicators in the country^18^. In 2019, the population under the jurisdiction of RHS/Diamantina was estimated to be 422, 578 habitants^19^. According to the Brazilian Notifiable Diseases Information System (SINAN-*Sistema de Informação de Agravos de Notificação*), the entire area reported 998 new cases of CL from 2005 to 2019.

### Study population

The study population comprised a non-probabilistic sample of patients with CL residing within the municipalities of RHS/*Diamantina.* All cases of CL reported to SINAN between January 2018 and December 2019 were considered. These individuals were diagnosed with CL confirmed by laboratory or clinical-epidemiological criteria and received specific treatment through systemic (IV-MA or IV-LAB) or intralesional (IL-MA) routes.

In systemic treatment with IV-MA, for cutaneous forms, it is recommended to administer 10-20 mg Sb+5/kg/ for 20 days, and for mucous forms, 20 mg of Sb5+/kg/ day for 30 days, preferably via slow IV injection and at rest after application, and a maximum of three ampoules per day. For IL-MA treatment, one to three subcutaneous applications of approximately 5 mL per session were administered, with an interval of 15 days. In IV-LAB, 2-5 mg/kg/day is recommended, with no maximum daily dose limit to reach a total dose of 25-40 mg/kg, by slow IV route and daily laboratory review of renal function, potassium, and magnesium serum^20^.

Individuals aged < 18 years, those who did not provide written consent, and those who did not answer either one or both questionnaires were excluded.

### Data collection

Data were collected retrospectively between February and October 2020. The patients were interviewed face-to-face during home visits or at the BCU in their territory of residence. The interviews were conducted using two questionnaires administered sequentially during the same interview by the research group or previously trained health professionals.

The first questionnaire (Supplementary Material) was semi-structured with questions addressing sociodemographic characteristics (i.e.,sex, age, area of residence, schooling level, occupation, and family income), existence of comorbidities, and access to and use of health services for the diagnosis and treatment of CL(i.e.,time between the appearance of the cutaneous lesion and health care seeking, first health service sought after the appearance of the cutaneous lesion, diagnosis of CL confirmed in the first health service sought, type of health service where the diagnosis of CL was confirmed, approximate distance between the patient’s household and the health service where the diagnosis of CL was confirmed, type of treatment, provision of information about possible adverse effects during treatment, occurrence of complications or adverse effects during treatment, approximate distance from the patient’s household to the health service where the treatment was performed, interruption of work/study activities, and additional costs incurred during diagnosis and/or treatment). 

The second questionnaire was the Cutaneous Leishmaniasis Impact Questionnaire (CLIQ)^16^. Briefly, the CLIQ is a psychometric questionnaire composed of 25 items distributed across two subscales:1) the general impact of CL and 2) patient perceptions of treatment and health services. The score for each item ranges from 0 to 4, with a maximum score of 100 points. Of these, 72 points refer to subscale 1 that are directly interpretable; the higher the score, the greater the general impact of the CL. Subscale 2 corresponds to 28 points that are indirectly interpretable; the lower the score, the greater the patient's satisfaction with treatment and health services^16^. CLIQ is available at https://doi.org/10.1371/journal.pone.0203378.s002.

### Data analysis

Data were coded in the Epi Info 7 software^21^ and analyzed using R 4.0.0 software^22^. Absolute and relative frequencies were calculated to describe categorical variables, and central tendency and dispersion to describe continuous variables.

Our main hypothesis was that the perception of the general impact of CL and patient satisfaction with treatment and health services measured by the CLIQ differed among patients who underwent different therapeutic approaches. Thus, we used the Kruskal-Wallis test to compare the CLIQ scores of patients treated with IV-MA, IV-LAB, and IL-MA for each CLIQ subscale. Differences were considered statistically significant at *P*< 0.05.

In addition, as proposed by Galvão et al.^16^, the median of scores obtained on the CLIQ subscales was used to dichotomize the general impact of CL (high vs. low impact) and patient perceptions of treatment and health services (low vs. high satisfaction). The association of high-impact and low-satisfaction outcomes with potential categorical predictors related to sociodemographic characteristics, existence of comorbidities, and access and use of health services for the diagnosis and treatment of CL was assessed in a univariate analysis using the chi-square test. For the low-satisfaction outcome, the general impact variable was also tested as a predictor. 

All variables with *P* ≤ 0.20 and expected frequency values > 5 were selected for multivariate logistic regression analysis. We developed adjusted models for each outcome (i.e.,high impact and low satisfaction) using a stepwise forward approach. The Akaike information criterion was employed to verify the effect of adding the predictors and interaction terms to the model fit. In both final models, we retained variables with *P*<0.05 and those relevant to improve the model fit. The Hosmer-Lemeshow test was performed to assess the goodness-of-fit of the models. We also checked for multicollinearity among the predictors. In both the univariate and multivariate analyses ,the strength of the association was determined using the odds ratio (OR) with a 95% confidence interval (95% CI).

### Ethical aspects

This study was approved by the Ethical Committee for Human Research of the Federal University of Jequitinhonha and Mucuri Valleys (CAAE number 25831919.0.0000.5108). Prior to enrolment, all patients were instructed about the research objectives, risks and benefits of participating, and the guarantee of anonymity. Written informed consent was obtained from all the patients. The procedures followed were in accordance with the ethical standards of the responsible committee on human experimentation and the principles of the Declaration of Helsinki, 1964, as revised in 1975, 1983, 1989, 1996, and 2000.

## RESULTS

Between 2018 and 2019, 146 CL cases were reported in the study area and were, therefore, potentially eligible to participate in the study. Of these, 39 (26.7%) relocated or were not accessible for data collection, 12 (8.2%) refused to participate, 7 (4.9%) did not answer both questionnaires, and 4 (2.8%) died of other causes. This resulted in 84 enrolled patients (57.5% of all notifications), all of whom presented with the cutaneous form of CL. All patients progressed to cure at the end of treatment; however, one patient treated with IV-LAB relapsed after the initial regimen and required a second regimen.

Most participants were male 43 (51.2%) and rural residents 65 (77.4%). The mean (standard deviation) age of the patients was 49.7 (17.0%) years, and the most frequent age group was ≥ 60 years 25 (29.8%). Regarding scholing level, 41 (48,8%) had completed primary scholl and 14 (16.7%) were illiterate. Farming 26 (31%) and retired status 15 (17.9%) constituted the predominant occupational status of the patients. Most individuals reported a family income between 1 and 3 Brazilian minimum wages 49 (58.3%), although a substantial proportion reported an income lower than 1 minimum wage 27 (32.1%). Almost half of the patients had comorbidities 39 (46.4%). Systemic arterial hypertension 26 (31%) was the most common comorbidity (Table 1).

**TABLE 1:** Sociodemographic characteristics and comorbidity status of individuals treated for cutaneous leishmaniasis in the municipalities under the jurisdiction of the Regional Health Superintendence of Diamantina, Minas Gerais State, Brazil, 2018-2019.

Variable	IV-MA	%	IL-MA	%	IV- LAB	%	All =84	%
**Sex**								
Male	19	50	12	44.4	12	63.2	43	51.2
Female	19	50	15	55.6	7	36.8	41	48.8
**Age (years)**								
18-20	1	2.6	1	3.7	0	0.0	2	2.4
20-30	4	10.5	3	11.1	0	0.0	7	8.3
30-40	12	31.6	5	18.5	0	0.0	17	20.2
40-50	17	44.7	1	3.7	1	5.3	19	22.6
50-60	3	7.9	5	18.5	6	31.6	14	16.7
≥ 60	1	2.6	12	44.4	12	63.2	25	29.8
**Area of residence**								
Rural	28	73.7	22	81.5	15	78.9	65	77.4
Urban	10	26.3	5	18.5	4	21.1	19	22.6
**Schoolinglevel**								
Illiterate	5	13.2	4	14.8	5	26.3	14	16.7
Primaryschool	17	44.7	13	55.6	11	68.4	41	48.8
High school	15	39.5	7	18.5	3	5.3	25	2.8
College	1	2.6	3	11.1	0	0.0	4	4.8
**Occupation**								
Retired	3	7.9	8	29.6	4	21.0	15	17.9
Hoseuwife	5	13.1	2	7.4	1	5.3	8	9.5
Farmer	14	36.8	6	22.2	6	31.6	26	30.9
Notreported	6	15.8	2	7.4	4	21.0	12	14.3
Others^a^	10	26.3	9	33.3	4	21.0	23	27.4
**Family income (Brazilian minimum wages)** ^b^								
< 1	15	39.5	5	18.5	7	36.8	27	32.1
1-3	21	55.3	17	63.0	11	57.9	49	58.3
3-5	2	5.3	2	7.4	1	5.3	5	6.0
≥ 5	0	0.0	3	11.1	0	0.0	3	3.6
**Comorbidity** ^c^								
Systemic arterial hypertension	8	22.9	11	39.3	7	33.3	26	31.0
Diabetes mellitus	1	2.9	2	7.1	4	19.0	7	8.3
Mental disorders	2	5.7	2	7.1	1	4.8	5	5.9
Cancer	0	0.0	0	0.0	1	4.8	1	1.2
None	24	68.6	13	46.4	8	38.1	45	53.6

^a^Unemployed (n = 4), self-employed (n = 3), general helper (n = 2), attendant (n = 2), foreman (n = 2), topography assistant (n = 1), mid (n = 1), course instructor (n = 1),mechanic (n = 1), machine operator (n = 1), civil servant (n =1), dentist (n = 1), accountant (n = 1), and veterinarian (n = 1). ^b^Brazilian minimum wage (2020): US$ 201.4 (R$ 1,045). ^c^Eighteen individuals reported more than one comorbidity. **CL:** cutaneous leishmaniasis; **IV-LAB:** intravenous liposomal amphotericin B; **IV-MA:** intravenous meglumine antimoniate; **IL-MA:** intralesional meglumine antimoniate.

Only 26 (31%) of the individuals sought healthcare within the first month they perceived the cutaneous lesion. Basic care units were the most frequently visited health service after the appearance of the lesion 59 (70.2%). However, only 33 (39.3%) of patients were diagnosed with CL during their first health service. Almost all CL cases were confirmed by public health services 76 (90.5%). Although diagnostic confirmation was mainly achieved in the municipality of residence 68 (81.0%), most patients 55 (65.5%) had to cover more than 10 km to reach the health service where they received confirmation (Table 2).

CL treatment was performed more frequently with IV-MA 38 (45.2%), followed by IL-MA 27 (32.1%). Complications or adverse effects were reported by 38 (45.2%) of patients, but only 22 (26.2%) were previously informed about the possibility of such events. Most individuals covered distances greater than 10 km to be treated 66 (78.5%), and half (n = 42) interrupted work and study activities at least once because of appointments related to CL or their treatment. Additional costs of CL diagnosis/treatment were reported by 63 (75%) of the patients, mainly due to transportation, laboratory exams, and food expenses (Table 2).

**TABLE 2:** Access and use of health services for the diagnosis and treatment of cutaneous leishmaniasis reported by patients from the municipalities under the jurisdiction of the Regional Health Superintendence of Diamantina, Minas Gerais State, Brazil, 2018-2019.

Variable	IV-MA	%	IL-MA	%	IV- LAB	%	All =84	%
**Time between the appearance of the cutaneous lesion and the health care seeking (months)**								
< 1	9	23.7	7	25.9	10	52.6	26	31
1-2	10	26.3	11	40.7	1	5.3	22	26
2-3	12	31.6	5	18.5	3	15.8	20	24
3-4	3	7.9	1	3.7	1	5.3	5	5.9
≥ 4	4	10.5	3	11.1	4	21.1	11	13
**First health service sought after the appearance of the cutaneous lesion**								
Basic care unit	25	65.8	21	77.8	13	68.4	59	70
Referral hospital	7	18.4	2	7.4	1	5.3	10	12
Polyclinic	4	10.5	1	3.7	4	21.1	9	11
Private doctor	2	5.3	3	11.1	1	5.3	6	7.2
**Diagnosis of CL confirmed in the first health service sought**								
Yes	14	36.8	9	33.3	10	52.6	33	39
No	24	63.2	18	66.7	9	47.4	51	61
**Type of health service where the diagnosis of CL was confirmed**								
Public	35	92.1	5	18.5	19	100	76	91
Private	3	7.9	22	81.5	0	0	8	9.5
**Diagnosis of CL confirmed in the municipality of residence**								
Yes	30	78.9	19	70.4	19	100	68	81
No	8	21.1	8	29.6	0	0	16	19
**Approximate distance between the patient’s household and the health service where the diagnosis of CL was confirmed (km)**								
< 1	5	13.2	2	7.4	4	21.1	11	13
1-5	6	15.8	2	7.4	3	15.8	11	13
5-10	17	44.7	10	37	8	42.1	7	8.3
10-30	4	10.5	3	11.1	1	5.3	35	42
30-100	1	2.6	4	14.8	2	10.5	8	9.5
≥ 100	5	13.2	6	22.2	1	5.3	12	14
**Provision of information about possible adverse effects during treatment**								
Yes	9	31	6	28.6	7	58.3	22	26
No	29	100	21	100	12	100	62	74
**Occurrence of complications or adverse effects during treatment** ^a^								
Yes	16	42.1	11	40.7	11	57.9	38	45
No	22	57.9	16	59.3	8	42.1	46	55
**Approximate distance from the patient’s household to the health service where the treatment was performed (km)**								
< 1	3	7.9	0	0	1	5.3	4	4.8
1-5	6	15.8	1	3.7	1	5.3	8	9.6
5-10	3	7.9	2	7.4	1	5.3	6	7.1
10-30	15	39.5	13	48.1	9	47.4	37	44
30-100	6	15.8	2	7.4	4	21.1	12	14
≥ 100	5	13.2	9	33.3	3	15.8	17	20
**Interruption of work/study activities during diagnosis and/or treatment**								
Yes	18	47.4	12	44.4	12	63.2	42	50
No	20	52.6	15	55.6	7	36.8	42	50
**Additional costs incurred during diagnosis and/or treatment**								
Yes	33	86.8	20	74.1	10	52.6	63	75
No	5	13.2	7	25.9	9	47.4	21	25

^a^ Most mentioned: weakness, vomiting, diarrhea, loss of appetite, weight loss, and laboratory abnormalities. **CL:** cutaneous leishmaniasis; **IV-LAB:** intravenous liposomal amphotericin B; **IV-MA:** intravenous meglumine antimoniate; **IL-MA:** intralesional meglumine antimoniate.

**FIGURE 1:**
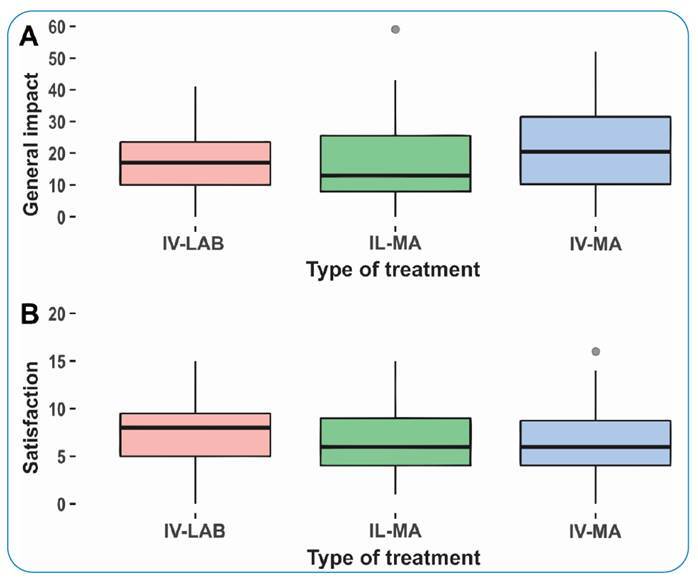
Scores on the general impact of cutaneous leishmaniasis (CL) **(A)** (*P*=0.455) and patient satisfaction with the treatment and health services **(B)** (*P*=0.447) measured by the subscales of the Cutaneous Leishmaniasis Impact Questionnaire^16^ among patients undergoing different therapeutic approaches for CL in the municipalities under the jurisdiction of the Regional Health Superintendence of *Diamantina*, *Minas Gerais* State, Brazil, from 2018 to 2019. **IV-LAB:** intravenous liposomal amphotericin B; **IV-MA:** intravenous meglumine antimoniate; **IL-MA:** intralesional meglumine antimoniate.

The general impact of CL and patient perceptions of treatment and health services defined by the CLIQ subscales presented an overall median (minimum-maximum) of 18 (0-59) and 6 (0-16) points, respectively. Patients treated with systemic therapies (medians: 20.5 and 17 points for IV-MA and IV-LAB, respectively) perceived a greater general impact of CL than that of those treated with intralesional administration (IL-MA: 13 points); however, this difference was not significant (*P* = 0.455) (Figure 1A). Regarding patient satisfaction with treatment and health services, individuals treated with IV-LAB (median: 8 points) showed lower satisfaction than that of those treated with IV-MA (median: 6 points) and IL-MA (median: 6 points); however, the difference was not statistically significant (*P* = 0.447) (Figure 1B).

The results of both the univariate and multivariate analyses of the factors associated with high CL impact, low treatment, and health service satisfaction are summarized in Tables 3 and 4 respectively. The high impact of CL was associated with low family income (OR:3.3; 95%CI:1.0-10.3), occurrence of complications or adverse effects during treatment (OR:7.7; 95%CI:2.4-25.6), and additional costs incurred during diagnosis and/or treatment (OR: 12.1; 95%CI:2.8-52.4) (Table 3). Low satisfaction with treatment and health services was associated with a high impact of the disease (OR: 9.5; 95% CI: 2.7-33.9), occurrence of complications or adverse effects during treatment (OR:4.2; 95%CI:1.3-13.0), and high family income (OR:7.1; 95%CI:1.7-28.2). The final model for low satisfaction was adjusted for the effect of hospitalization (Table 4).

**TABLE 3:** Factors associated with the high impact of cutaneous leishmaniasis among patients from the municipalities under the jurisdiction of the Regional Health Superintendence of Diamantina, Minas Gerais State, Brazil, 2018-2019.

	General impact of TL				(95% CI)		(95% CI)	
Variable	High		Low		Crude OR	*P*	Adjusted OR	*P*
	n	%	n	%				
**Sex**								
Male	20	46.5	23	53.5	0.6 (0.2-1.6)	0.380	-	-
Female	23	56.1	18	43.9	1		-	-
**Age group (years)** ^a^								
<48	22	53.4	20	47.6	1	0.827	-	-
≥ 48	21	50.0	21	50.0	0.9 (0.3-2.1)		-	-
**Area of residence**								
Rural	34	52.3	31	47.7	1.2(0.4-3.3)	0.705	-	-
Urban	9	47.4	10	52.6	1		-	-
**Schooling level**								
Illiterate - Primary school	29	52.7	26	47.3	1.1 (0.4-2.9)	0.698	-	-
High school - College	14	48.3	15	51.7	1		-	-
**Family income (Brazilian minimum wages)** ^b^								
< 1	18	67.7	9	33.3	2.5 (0.9-6.6)	0.051	3.3 (1.0-10.3)	0.039
≥ 1	25	43.9	32	56.1	1		1	-
**Comorbidity**								
No	21	46.7	24	53.3	1	0.373	-	-
Yes	22	56.4	17	43.6	1.4 (0.6-3.5)		-	-
**Diagnosis of CL confirmed in the first health service sought**								
Yes	15	51.4	17	48.6	1	0.535	-	-
No	28	53.8	24	46.2	1.3 (0.5-3.1)		-	-
**Approximate distance between the patient’s household and the health service where the diagnosis of CL was confirmed (km)**								
< 10	16	55.2	13	44.8	1	0.596	-	-
≥ 10	27	49.1	28	50.9	0.7 (0.3-1.9)		-	-
**Type of treatment**								
Intralesional^c^	12	44.4	15	55.6	1	0.395	-	-
Systemic^d^	31	54.4	26	45.6	1.4 (0.5-3.7)		-	-
**Occurrence of complications or adverse effects during treatment**								
No	16	35.6	29	64.5	1	<0.001	1	0.001
Yes	27	69.2	12	30.8	4.0 (1.6-10.1)		7.7 (2.4-25.6)	
**Approximate distance from the patient’s household to the health service where the treatment was performed (km)**								
< 10	10	55.6	8	44.4	1	0.676	-	-
≥ 10	33	50.0	33	50.0	0.8 (0.2-2.2)		-	-
**Hospitalization during treatment**								
No	26	47.2	29	52.7	1	0.323	-	-
Yes	17	58.6	12	41.4	1.5 (0.6-3.9)		-	-
**Additional costs incurred during diagnosis and/or treatment**								
No	4	21.1	15	78.9	1	0.003	1	0.001
Yes	39	60.0	26	40.0	5.6 (1.6-18.8)		12.1 (2.8-52.4)	
**Interruption of work/study activitiesduring diagnosis and/or treatment**								
No	15	38.5	24	61.5	1	0.030	-	-
Yes	28	62.2	17	37.8	2.6 (1.0-6.3)		-	-

**OR:** odds ratio; **95% CI:** confidence interval at 95%; **CL:** cutaneous leishmaniasis. ^a^Age variable categorized by the median. ^b^Brazilian minimum wage (2020): US$ 201.4 (R$ 1,045). ^c^Intralesional administration of meglumine antimoniate. ^d^Intravenous administration of meglumine antimoniate or liposomal amphotericin B.

**TABLE 4:** Factors associated with the low satisfaction with treatment and health services among patients treated for cutaneous leishmaniasis from the municipalities under the jurisdiction of the Regional Health Superintendence of Diamantina, Minas Gerais State, Brazil, 2018-2019.

	Satisfaction with treatment and health services							
	High		Low		Crude OR (95% CI)	*P*	Adjusted OR (95% CI)	*P*
Variable	n	%	n	%				
**Sex**								
Male	26	60.4	17	39.6	1	0.194	-	-
Female	19	46.3	22	53.7	0.5 (0.2-1.3)		-	-
**Age group (years)** ^a^								
<48	23	54.8	19	45.2	1.1 (0.4-2.5)	0.827	-	-
≥ 48	22	52.4	20	48.6	1		-	-
**Area of residence**								
Rural	37	54.4	28	45.6	1	0.255	-	-
Urban	8	42.1	11	57.9	0.5 (0.1-1.5)		-	-
**Schooling level**								
Illiterate - Primary school	33	60.0	22	40.0	1	0.104	-	-
High school - College	12	41.4	17	58.6	0.4(0.1-1.1)		-	-
**Family income (Brazilian minimum wages)** ^b^								
<1	18	66.7	9	33.3	2.22 (0.8-5.7)	0.101	7.09 (1.7-28.2)	0.005
≥ 1	27	47.4	30	52.6	1		1	
**Comorbidity**								
Yes	22	56.4	17	43.6	1	0.627	-	-
No	23	51.1	22	48.9	0.8 (0.3-1.9)		-	-
**Diagnosis of CL confirmed in the first health service sought**								
Yes	18	56.2	14	43.8	1.1 (0.4-2.8)	0.699	-	-
No	27	51.9	25	48.1	1		-	-
**Approximate distance from the patient’s household to the health service where the diagnosis of CL was confirmed (km)**								
< 10	16	55.2	13	44.8	1	0.830	-	-
≥ 10	29	52.7	26	47.3	0.9 (0.4-2.2)		-	-
**Type of treatment**								
Intralesional^c^	14	51.9	13	48.1	1	0.828	-	-
Systemic^d^	31	54.4	26	45.6	1.1 (0.4-2.7)		-	-
**Occurrence of complications or adverse effects during treatment**								
Yes	12	30.8	27	69.2	1	<0.001	1	0.013
No	33	73.3	12	26.7	6.1 (2.3-15.9)		4.2 (1.3-13.0)	
**Approximate distance between the patient’s household and the health service where the treatment was performed (km)**								
< 10	9	50.0	9	50.0	1	0.732	-	-
≥ 10	36	54.5	30	45.5	0.8 (0.2-2.3)		-	-
**Hospitalization during treatment**								
Yes	10	34.5	19	65.5	1	0.011	1	0.061
No	35	63.7	20	37.7	3.3 (1.2-8.5)		3.2 (0.9-10.9)	
**Additional costs incurred during diagnosis and/or treatment**								
Yes	33	50.8	32	49.2	1	0.341	-	-
No	12	63.2	7	36.9	1.6 (0.5-4.7)		-	-
**Interruption of work/study activities during diagnosis and/or treatment**								
Yes	25	55.6	20	44.4	1	0.695	-	-
No	20	51.3	19	48.7	0.8 (0.3-1.9)		-	-
**General impact of CL**								
High	14	32.6	29	66.4	1	<0.001	1	<0.001
Low	31	75.6	10	24.3	6.4 (2.4-16.7)		9.5 (2.7-33.9)	

**OR:** odds ratio; **95% CI:** confidence interval at 95%; **CL:** cutaneous leishmaniasis. ^a^Age variable categorized by the median. ^b^Brazilian minimum wage (2020): US$ 201.4 (R$ 1045). ^c^Intralesional administration of meglumine antimoniate. ^d^Intravenous administration of meglumine antimoniate or liposomal amphotericin B.

## DISCUSSION

The impact of CL on patient quality of life has been minimally explored in the literature^17^. In addition, limited Brazilian data on intralesional therapy are available, which could explain that this treatmentis not the first choice. Our study explored this impact using a specific questionnaire for CL, which emphasized the perception of treatment and health services offered to patients in a Brazilian region endemic to the disease. Although we did not detect significant differences in the perceived impact of CL and satisfaction with treatment and health services among individuals treated with IL-MA or systemic therapies, the medians of CLIQ scores were substantially different between the groups. This discrepancy is likely a consequence of the simplification, shorter duration, fewer visits to health services, and greater clinical safety provided by intralesional therapy compared with that of systemic therapies^7^ ^,^ ^23^. Notably, our sample size and selectionmay have introduced a type II error.

Additionally, despite the direct costs of CL treatment being covered by the Brazilian Unified Health System, long therapeutic regimens usually increase the impact of the disease by demanding additional costs and causing loss of work and study opportunities for patients and their relatives^24^. A study of Bolivian patients demonstrated that treatment of CL with IL-MA caused a relatively reduced loss of work and cost compared with that of IV-MA therapy^25^. We found that patients treated with IV-LAB were less satisfied with therapy and health services than that of those receiving other treatments. This finding may be related to the need for hospitalization for IV-LAB administration, which alters patients' daily routines^26^.

We also demonstrated that both low satisfaction with treatment and high impact of CL were associated with the occurrence of complications and adverse effects. Therefore, adverse reactions related to the administration of IV-MA, the most common therapeutic approach in this study, must be considered. The side effect profile of IV-MA therapy is broad, ranging from mild but uncomfortable events, such as musculoskeletal pain, gastrointestinal disturbances, and headache, to severe side effects, such as prolonged electrocardiographic QT interval and acute pancreatitis^27^.

Family income was also identified as a factor associated with the outcomes of the present study, although in the reverse relationship. Individuals with high income perceived lower levels of satisfaction with treatment and health services. Martins et al.^28^reported a similar relationship when investigating the negative evaluations of dental services in Brazil. The assessment of health services has been shown to be unequal between groups and social classes depending on individual socioeconomic status^29^ ^,^ ^30^. The relatively more critical perceptions displayed by individuals of higher socioeconomic status are different from the feelings of resignation and fatalism observed in individuals with lower socioeconomic status^29^ ^,^ ^30^.

On the other hand, we observed that individuals with lower incomes perceived themselves as being more affected by the disease. Given that patients with CL mostly reside away from health facilities and/or in rural locations, it is plausible to infer that their diagnosis and treatment incurred a substantial indirect financial burden, which may have compromised their limited family budgets^31^.The additional costs incurred by the patients were also associated with a negative perception of CL. Similarly, a study carried out with patients treated at a referral center in the Brazilian state of Minas Gerais found that disease-related expenses above US$ 137 significantly impacted the patient’s quality of life^31^. The authors also demonstrated that dissatisfaction with financial resources among individuals treated for CL negatively impacted their quality of life^32^.

The main limitation of the present study was the inability to generalize the results because of the small sample size and non-probability. Furthermore, these findings may not be extrapolated to mucocutaneous leishmaniasis, as no patients with this clinical presentation were enrolled in our investigation. Despite this, our results provide direction for improving public policies aimed at CL management. Given the difficulty of accessing health services encountered by affected populations, it is strongly recommended that IL-MA be administered in primary healthcare services whenever clinically indicated. This approach is beneficial both at the individual level and from a public health perspective as it is simpler, more practical, safer, highly acceptable, and cost-effective^13^.

Thus, better structuring of healthcare networks and continuous professional training focused on the timely detection and treatment of leishmaniasis should be implemented. In parallel, studies aimed at more comfortable and safer therapies and new drugs with oral formulations should be encouraged for the treatment of CL. Finally, public policies to improve the quality of life of the affected populations in endemic areas are of paramount importance. These strategies can reduce the impact of CL and increase patient satisfaction with treatments and health services.

Although we did not detect significant differences in perceived impact and treatment satisfaction among patients undergoing different therapeutic approaches in the study area, we believe that further studies in other endemic areas will contribute to providing an overview of this issue, as intralesional therapy has recently been introduced as a routine. We observed that the perceived high impact of CL was associated with the occurrence of complications or adverse effects during treatment, additional costs, and lower income. Low satisfaction with treatment and health services was associated with a high impact of the disease, complications, or reactions during treatment, and high family income.As evidence of good intralesional therapy efficacy grows, results such as those presented here should be considered to define more viable and feasible treatments.
