# Supplementary material for: “It’s not all about the disease”: do treatment and socioeconomic status affect perceived impact and satisfaction of patients treated for cutaneous leishmaniasis?
Source: Rev Soc Bras Med Trop. 2023 Feb 20;56:e0253-2022. doi: 10.1590/0037-8682-0253-2022 (PMC9957146; doi:10.1590/0037-8682-0253-2022)
Supplement: Supplementary file 1 [file 1678-9849-rsbmt-56-e0253-2022-supp1.pdf]

QUESTIONNAIRE

1.PERSONAL DATA

Name: ____

City of residence: ______________________________________

Area of residence: ( )Urban ( )Rural

Date of birth: ____/______/____**Interview date**:____/_____/_____

Sex: ( )Female ( ) Male

Education:

( )Illiterate

( ) Incomplete primary education

( ) Completed primary education

( ) Incomplete elementary education

( ) Completed elementary education

( ) Incomplete secondary education

( )Completed secondary education

( )Graduate

Occupation. Specify:_____________________________________

How many people live in the same household as you? ____________

Who are these people? ( ) Partner ( ),Childrenunder 12 years of age ( ),Parents

( )Others. Specify:____

What is the monthly family income?

( ) <1 Brazilian minimum wage (upto R$ 998.00)

( ) 1 to 3 Brazilian minimum wages (from R$ 998.00 to R$ 2.294)

( ) 3 to 5 Brazilian minimum wages (from R$ 2.295 to 4.990)

( ) > 5 Brazilian minimum wages (more than R$ 4.990)

Do you have health insurance? ( ) Yes ( ) No

If yes, did you use the health insurance for medical appointments or laboratory tests related to cutaneousleishmaniasis (CL)? ( ) Yes ( ) No

Do you suffer from any disease other than CL? ( ) Yes ( ) No

If yes, specify:

( ) Diabetes mellitus

( ) Hypertension (high blood pressure)

( )Dyslipidemia (high cholesterol level)

( ) Chagas disease

( ) Other:____

Are you currently under going any medical treatment for these diseases or do you use any medication regularly? ( ) Yes ( ) No

2 ASSISTANCE ASPECTS

How far is your home from the nearest health facility?

( )<1 km

( ) Between 1 and 5 km

( ) Between 5.1 and 10 km

( ) Between 10.1 and 30 km

( )Between 30.1 and 100 km

( )> 100 km

What means of transport did you use to go to the health facility?

( )None. Go on foot

( )Bicycle

( )Car

( ) Bus

( )Other. Which one?______________________

How long does it take to get from your home to the nearest health center?

( )< 15 minutes

( )from 15 to 30 minutes

( )from 30 to 60 minutes

( ) from 1 to 2 hours

( )> 2 hours

After you noticed the wound, how long did you seek the health service?

( ) During the month in which the lesion appears

( ) 1 month later

( ) Between 2 and 3 months

( ) Between 3 and 4 months

( ) > 4 months

( ) Other. Specify when?_____________________

What was the first health service you sought after noticing the wound?__________

Did you need another health service after ward? ( ) Yes ( )No

How many?____________________Which one?______________________

Were you diagnosed with CL at the first health service you sought? ( ) Yes ( ) No

If not, how many health services did you have to go to until your CL was diagnosed?

( ) 1 ( ) 2 ( ) 3 ( ) 4 ( ) 5 ( ) > 5 ( ) I do not know

Which one?____

Where was the diagnosis of CL confirmed?

( ) In the municipality where you live

( ) In another municipality, specify__________

What is the approximate distance between your home and the place where the diagnosis of CL was confirmed?

( )> 1 km

( ) Between 1 and 5 km

( ) Between 5.1 and 10 km

( ) Between 10.1 and 30 km

( )Between 30.1 and 100 km

( )< 100 km

The diagnosisof CL was confirmed in which type of health service?

( )Public ( ) Private

Where was the diagnosis confirmed?

( )At the basic health care unit in the area of residence

( )Another basic health care unit

( )At the out patient clinic of UFVJM,Faculty of Medicine.

( )René Rachou (Belo Horizonte)

( )Hospital

( )Private doctors

( )Other Locations. Specify:________________________________

Where was the laboratory test performed thatconfirmed CL?

( )Municipal laboratories

( ) Laboratory of Diamantina Regional Health Superintendence

( ) Laboratory of the Federal University of Jequitinhonha and Mucuri Valleys.

( ) René Rachou

( )Private laboratories

( )Other. Specify:____

How many times did you have to leave your home dueto CL?

For treatment: ________For appointments: ________

What is the approximate distance between your home and the place where you received CL treatment?

( )< 1 km

( ) Between 1 and 5 km

( ) Between 5.1 and 10 km

( ) Between 10.1 and 30 km

( )Between 30.1 and 100 km

( )> 100 km

What means of transportation do you use/did you use to travel to the place where you received CL treatment? (there is more than one answer option)

( )By foot

( ) Bus

( )Intercity buses

( )Sanitary car/city hall transport

( )Own car

( ) Taxi

( )Others. Specify: ___________________________

Has any one who lives with you ever had CL ( ) Yes ( ) No

If yes, howmany?_______________

Type of treatment:

( )Intralesional

( ) Systemic glucantime

( ) Systemic liposomal amphotericin B

( )Other. Specify:__________________________________

How long did the treatment last?

( ) Between 1 and 20 days

( ) Between 21 and 30 days

( ) Between 31 and 60 days

( ) Between 61 and 90 days

( ) Between 90 and 120 days

( )> 120 days. How much?________________

How long did it take for the wound to close?

( ) Up to month

( ) Between 1 and 2 months

( ) Between 2 and 3 months

( ) Between 3 and 4 months

( ) Between 4 and 5 months

( ) Between 5 and 6 months

( )> 6 months. How much?____________________________

Have you ever forgotten to take the drug for the treatment of CL? ( ) Yes ( ) No

Have you ever stopped taking the drug for the treatment of CL because you thought you were already feeling better? ( ) Yes ( ) No

Have you ever stopped taking the drug for the treatment of CL, thinking that it was making you sick?

( ) Yes ( ) No

Were you informed about the possible adverse effects of the drug used for the treatment of CL?

( ) Yes ( ) No ( ) I do not know

Did you have any reaction caused by the drug used for the treatment of CL?

( ) Yes ( )No ( ) I do not know

If the answeris YES, specify which ones:____________________________

____

Did you have any complications/side effects related to the treatment of CL?

( ) Yes ( ) No

If yes, specify:___________________________

Have you ever been hospitalized because of CL? ( ) Yes ( ) No

If so, how many times? _________How long were you hospitalized? _______

Were you submitted to any laboratory tests during the treatment of CL?

( )Yes ( ) No

If yes, which ones?______________________

Were you satisfied with the treatment of CL?

( ) Yes, totally ( ) Yes, partially ( ) No ( ) I do not know

3. ECONOMIC ASPECTS

Did you have extra expenses because of CL?( ) Yes ( ) No

What did you spend due to CL?

( ) Medical appointments.

( ) Hospital fee

( )Laboratory tests

( )Drugs

( )Transportation.

( )Food away from the home

( ) Caregivers (nanny) for children

( ) Employees who provide domestic services

( ) Employees performing job-related tasks

( ) Materials used for dressing

( ) Health insurance-relatedcosts

( ) Other expenses. Specify:______________________________

What was the estimated cost of spending? _______________________

Did you stop your work or study activities due to CL or its treatment?

( ) Yes ( ) No

If yes, due to CL? ( )Yes ( ) No If yes, for how many days?____

If yes, because of treatment with CL? ( )Yes ( )No If yes, for how many days?____
